# Corrective Osteotomy of Upper Extremity Malunions Using Three-Dimensional Planning and Patient-Specific Surgical Guides: Recent Advances and Perspectives

**DOI:** 10.3389/fsurg.2021.615026

**Published:** 2021-02-03

**Authors:** Babak Saravi, Gernot Lang, Rebecca Steger, Andreas Vollmer, Jörn Zwingmann

**Affiliations:** ^1^Department of Orthopedics and Trauma Surgery, Faculty of Medicine, Medical Centre, Albert-Ludwigs-University of Freiburg, Freiburg, Germany; ^2^Department of Oral and Maxillofacial Surgery, Faculty of Medicine, Medical Centre, Albert-Ludwigs-University of Freiburg, Freiburg, Germany; ^3^Department of Orthopedics and Trauma Surgery, St. Elisabeth Hospital Ravensburg, Ravensburg, Germany

**Keywords:** three-dimensional (3D), patient-specific 3D preoperative templating, surgical guides, corrective osteotomy, malunion, upper extremity (arm)

## Abstract

Malunions of the upper extremity can result in severe functional problems and increase the risk of osteoarthritis. The surgical reconstruction of complex malunions can be technically challenging. Recent advances in computer-assisted orthopedic surgery provide an innovative solution for complex three-dimensional (3-D) reconstructions. This study aims to evaluate the clinical applicability of 3-D computer-assisted planning and surgery for upper extremity malunions. Hence, we provide a summary of evidence on this topic and highlight recent advances in this field. Further, we provide a practical implementation of this therapeutic approach based on three cases of malunited forearm fractures treated with corrective osteotomy using preoperative three-dimensional simulation and patient-specific surgical guides. All three cases, one female (56 years old) and two males (18 and 26 years old), had painful restrictions in range of motion (ROM) due to forearm malunions and took part in clinical and radiologic assessments. Postoperative evaluation of patient outcomes showed a substantial increase in range of motion, reduction of preoperatively reported pain, and an overall improvement of patients' satisfaction. The therapeutic approach used in these cases resulted in an excellent anatomical and functional reconstruction and was assessed as precise, safe, and reliable. Based on current evidence and our results, the 3-D preoperative planning technique could be the new gold standard in the treatment of complex upper extremity malunions in the future.

## Introduction

**Figure 1:**
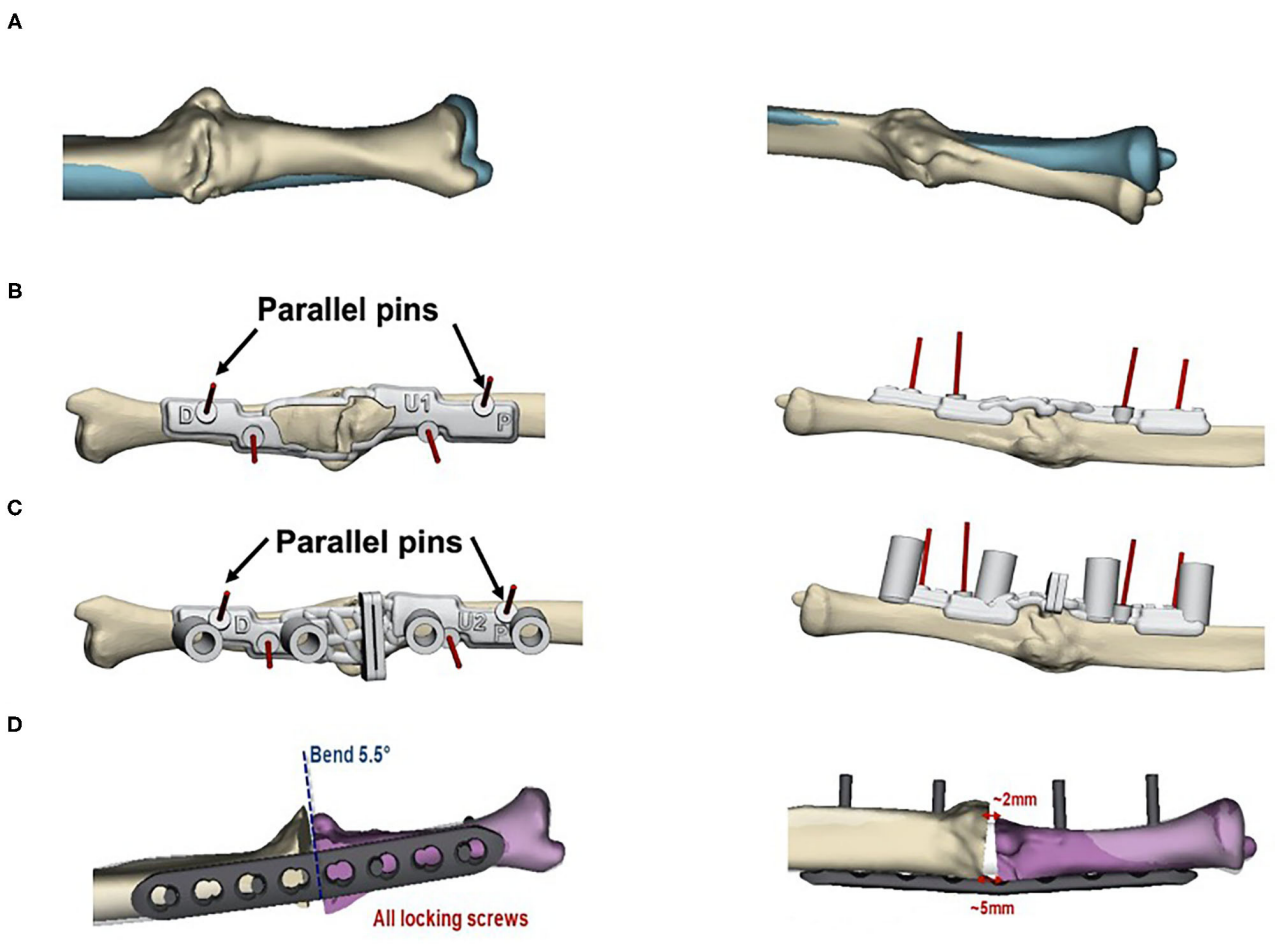
Preoperative planning for the corrective osteotomy. 3-D- planned corrective osteotomy of forearm malunions typically involves the following steps: (1) obtaining CT data from malunited and contralateral non-affected forearm (2) creation of virtual models of both forearms (3) construction of patient-specific surgical guides to transform the digital process into the actual surgery. Rapid prototyping of the digital bone models can be used to compare the fitting of the guides during surgery (11–14). The workflow is shown for case 2 in our study: **(A)** Preoperative situation with the contralateral bone template shown in blue. Construction of bone models was based on preoperative CT data of both forearms; **(B)** Bone marking guide to mark the area in the window for verification of the drilling and cutting guides' position; **(C)** Drilling and cutting guide affixed to the bone using K-wires; verification of the desired position can be made by comparison with the guide outline on the bone model; **(D)** Simulated outcome model with the size of the bone wedges and the bending angle of the plate osteosynthesis.

The forearm is a complex anatomical and functional unit, including the radius, the ulna, the interosseous membrane (IOM), the triangular fibrocartilage complex (TFCC), the proximal (PRUJ) and the distal (DRUJ) radioulnar joint. Forearm malunions represent a disruption of this functional system, which could lead to limited forearm range of motion, loss of power, visible cosmetic changes, painful forearm rotations due to bony impingement of the IOM, and instability of the DRUJ (1, 2). Further, the malunion can increase the risk for osteoarthritis, especially if intra-articular structures are involved (3, 4). A range of motion in pronation and supination of 50° is reported to be required for most daily activities. Malunions with rotational deformities lead to losses in the motion range of pronation and supination and thus, significantly affect the quality of life (1, 5). The anatomical and functional reconstruction of diaphyseal forearm fractures with compression plate and screw-fixation is a well-established surgical intervention with a relatively low complication rate (6–9). A more difficult surgical challenge is the reconstructive surgery of malunited fractures that may occur after conservative or operative treatment of the fracture (1). The multidimensional reconstruction of the axial alignment, the restoration of the normal length, and correction of angular deformities is a demanding challenge for the surgeon. Precise preoperative planning is known to be essential in the correction of complex diaphyseal malunions (2). However, it is very difficult to fully assess these deformities via two-dimensional x-ray images. Computer-aided planning based on preoperatively acquired computer tomography (CT) data allows the surgeon to accurately visualize the operation via computer simulation and to produce patient-specific surgical guides based on the three- dimensional imagery of patients' anatomy (2, 10). The workflow for the application of the 3-D planning technique is shown in Figure 1. Preoperative three-dimensional planning and use of patient-specific guides were already known for many years in dental implant surgery and total knee arthroplasty and there is clear evidence that highlight the benefits compared to 2D techniques (15–17). However, there is no summary of evidence regarding the applicability of three-dimensional preoperative planning techniques and application in the surgery of upper extremity malunions.

Here, we provide evidence from recent advances and present three cases of forearm malunions treated with corrective osteotomy using preoperative three-dimensional planning and patient-specific surgical guides to evaluate the outcomes of this innovative technique and close the present evidence gap.

## Three-Dimensional Virtual Planning of Corrective Osteotomies of Upper Extremity Malunions: Previous Studies

First studies on the application and accuracy of preoperative simulation and production of computer-designed bone models of the upper extremity based on patients' CT data were provided by the workgroup of Oka et al. (18) and Oka et al. (19). In 22 patients, including patients with malunited forearm fractures, malunited distal radial fractures, and cubitus varus deformity, preoperative simulation, and design of a custom made osteotomy template to reproduce the simulation during the actual surgery resulted in satisfying outcomes with an osseous union in all patients within 6 months and a full anatomical reconstruction of the angular deformities (20). Further, intra-articular fracture of the distal radius could be successfully treated through an extra-articular approach with this technique (19). The majority of studies in the literature since then focus on three-dimensional corrective osteotomy, particularly for distal radius fractures. In a systematic review and meta-analysis conducted by Keizer et al. (11), including 15 studies with a total of 68 patients, the authors concluded that preoperatively present palmar tilt, radial inclination, and ulnar variance in 96% of the observed cases with distal radius malunions could be significantly improved with the 3-D planning technique (11). The restoration reached 5° or 2 mm of their normal values. Despite these promising results, complications occurred in 11 out of 68 patients (16%) of the patients, including postoperative hardware-related pain or discomfort with subsequent hardware removal (*n* = 6), postoperative screw loosening (*n* = 2), a persistent distal radioulnar subluxation (*n* = 2), and partial laceration of the extensor pollicis longus tendon (*n* = 1). The authors argued that especially corrective osteotomies of distal radius malunions tend to show higher percentages of complications compared to less complex elective wrist surgery (11, 21). In a case series of four patients with combined intra- and extraarticular malunion of the distal radius, the accuracy of the correction quantified by comparing the virtual three-dimensional planning models with the corresponding postoperative 3-D bone model was −1° ± 5° for the radial inclination, 0 ± 1 mm for the ulnar variance, respectively (22). However, the volar tilt was under-corrected in all cases, with an average of −6 ± 6°. Corrective osteotomy of Monteggia lesions, in which the dislocation of the radial head is associated with a malunited fracture of the ulna, was examined in two case reports conducted by Oka et al. (23). The combined radius and ulnar fracture in both patients were initially treated non-operatively with closed reduction and casting, which resulted in malunion with chronic radial head dislocation. The preoperative planning and evaluation of the kinematics of joint motion and the use of CT bone models and surgical guides to transfer the simulation into the surgical situation resulted in a restoration of forearm rotation and absence of preoperatively reported elbow pain. 3-D corrective osteotomy in both forearm bones, ulna and radius, was performed by Miyake et al., reporting results from 14 patients with a median follow-up of 29 months (10). The authors reported excellent radiographic and clinical outcomes with an improvement of the mean arc of forearm motion from 76° preoperatively to 152° postoperatively, and improved grip strength from 82 to 94%, compared to that of the contralateral non-affected side.

One of the main advantages of three-dimensional corrective osteotomy is the preoperative assessment of the 3-D anatomy, which need to be taken into account for complex deformities such as cubitus varus or valgus deformities. This therapeutic approach allows the surgeon, therefore, to not only preferentially focus on the coronal plane but also precisely take into account internal rotation, flexion-extension deformities of the elbow, and lateral protrusion of the distal fragments in such complex deformities (24, 25). In a study conducted by Chung et al., 3-D corrective osteotomy of cubitus varus deformities in 23 adult patients resulted in an improvement of humeral-elbow-wrist angle from a mean of 26° preoperatively to a mean of 3° postoperatively, whereas the mean internal rotation angle improved from 25° to 5° (25). No recurrence of the deformity was observed in the averaged 1 year and 10 months follow-up period after osteotomy, and the authors recommended this technique with regards to these satisfactory outcomes. Outcomes of 3-Dimensional corrective osteotomy for supracondylar cubitus valgus, and cubitus varus deformities, respectively, and diaphyseal malunions were analyzed in a case series of nine patients conducted by Kataoka et al. (26). The error in corrective osteotomy between the preoperative simulation and postoperative bone model manufactured by rapid prototyping was <3 mm and 2°. Patients who experienced wrist pain before surgery reported a substantial decrease in pain after surgery. The authors rated this technique as precise, relatively easily performable, and satisfactory for the treatment of complex supracondylar malunions with regards to the clinical outcome. In another cohort study including 30 consecutive patients with a cubitus varus resulting from malunion of distal supracondylar fractures, the mean humerus-elbow-wrist angle and tilting angle improved significantly from 18.2° (varus), and 25°, respectively, preoperatively to 5.8° (valgus) and 38°, respectively, postoperatively (27). Preoperatively reported hyperextension of the elbow and internal rotation of the shoulder were normalized in all included patients, and the authors judged this technique to be a feasible treatment option for cubitus varus deformity. Finally, in a case series including 17 patients with cubitus varus deformities after supracondylar fractures, comparison of postoperative 3-D bone models with preoperative simulation revealed an improvement of 15° in varus before surgery to 6° in valgus after surgery, whereas the mean tilting angle of the affected side improved from 31° preoperatively to 40° postoperatively (28). The authors concluded that varus deformities could be corrected accurately with the 3-D corrective osteotomy technique. Overall, these studies provide evidence that three-dimensional corrective osteotomy techniques for complex upper extremity malunion, such as cubitus varus and valgus deformities, respectively, can accurately and reliably restore the normal anatomical condition.

## Preliminary Results From Three Cases

**Figure 2:**
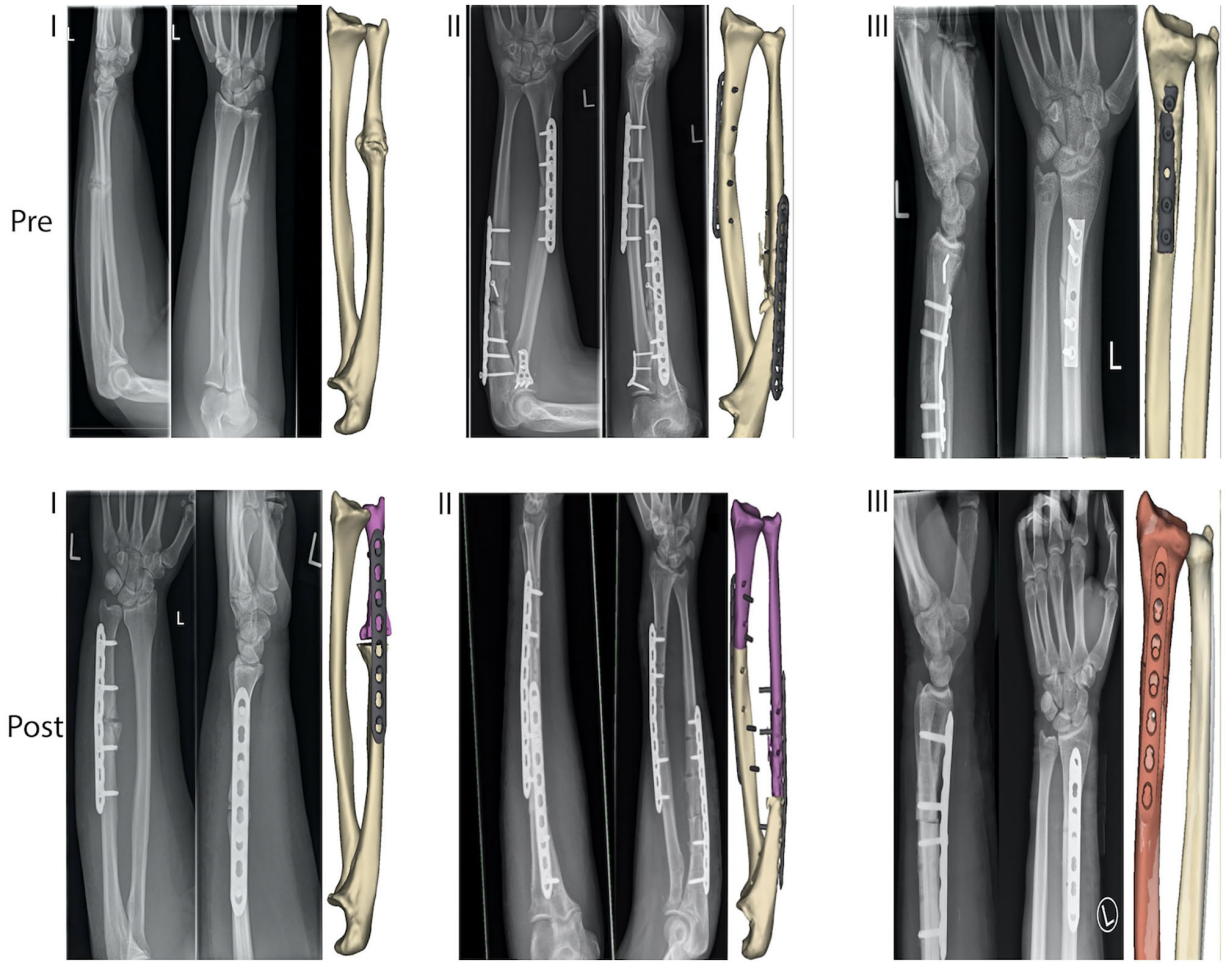
Preoperative (“Pre”) and postoperative (“Post”) simulation model and radiography of three cases (I., II., and III.), treated with three-dimensional preoperative planning and corrective osteotomy of the forearm malunions.

All three cases, one female (56 years old) and two males (18 and 26 years old), had painful restrictions in range of motion (ROM) due to forearm malunions and took part in clinical and radiologic assessments (Figure 2). Based on this situation, corrective osteotomy with three-dimensional planning and patient-specific guides was proposed (*Materialise* NV, Leuven, Belgium). Based on patients' CT data, three-dimensional bone models were created to assess the deformity and plan the corrective osteotomy. Malunions of the affected bones were further evaluated with the contralateral non-affected bone as a mirrored and precisely aligned ideal template for the correction. The planning of the corrective osteotomy was conducted with a clinical engineer to superimpose the contralateral template and simulate the corrective osteotomy. For both bones, ulna and radius, a closing osteotomy after removing a wedge of bone and consecutive plate fixation was simulated. According to the simulation, we determined the plate bending angle for the radius and the ulna, to fixate the fragments in the desired position, appropriately. One patient-specific drilling guide and one patient-specific combined drilling and cutting guide were designed for radius and ulna, respectively, to reproduce the simulation to the surgery. Guides' cutting slots were designed to orient the saw blade, and guiding holes served to orient K-wires as a reference during fragment reduction. Furthermore, drilling holes helped predrilling screw holes as an additional reference during fragment reduction and plate fixation. Fixation was performed with screws on each side of the osteotomy line. Follow-up of up to 9 months revealed a significant improvement of range of motion, which reached the level of the non-affected side in the first 2 months. The patients reported a significant improvement in pain and satisfaction compared to the preoperative situation. Fist closure and finger spreading were possible without restriction. No sensorimotor deficits were observed in clinical examination.

## Discussion

In the three presented cases, three-dimensional corrective osteotomy of forearm malunions resulted in excellent results. There is currently limited evidence from studies comparing three-dimensional surgical planning techniques with conventional planning methods with regards to forearm malunions to the best of our knowledge. One important reason is that three-dimensional planning techniques are often used for more complex cases, therefore making it difficult to compare cohorts (11). Vroemen et al. retrospectively investigated postoperative position after corrective osteotomy using two-dimensional vs. three-dimensional preoperative planning techniques of forearm malunions (29). They reported a significant correlation of malposition (assessed with malalignment parameters, such as palmar tilt, radial inclination, and ulnar variance) with the clinical outcome only for the three-dimensional rotational deficits but not between 2-D standard radiographs and the respective malalignment parameters. Therefore, this suggests that 3-D planning technique can more precisely assess misalignment and thus predict the clinical outcome to a higher degree compared to preoperative planning with conventional 2-D radiography. Buijze et al. recently published a randomized controlled trial comparing three-dimensional with two-dimensional preoperative planning techniques for corrective osteotomy of distal radius malunions (30). They reported a trend toward a minimal clinically important difference in patient-reported outcomes, such as pain and satisfaction scores in favor of the 3-D computer-assisted technique. However, the results were not significant, and the authors could not draw a definitive conclusion. According to these results, conventional surgical techniques and preoperative 2D fluoroscopy might be sufficient in many cases, but more complex cases involving combined intra- and extraarticular corrective osteotomies and multidimensional correction could be primarily managed by the 3-D technology in the future (22).

Patient-specific guides based on preoperative three-dimensional techniques have been reported to be accurate and easier to handle, and integration into the surgical workflow is simple to implement with structured training and the help of clinical engineers (12, 31). Another advantage is that additional conventional bending of the plates is avoided as the bending angle can be preoperatively simulated and therefore be reproduced during surgery. This leads to a reduced mean operative time and may reduce intraoperative bleeding, thus leading to a better functional recovery, as suggested previously (32). Errors of <1° and 1 mm for simulated osteotomies have been described for osteotomies with patient-specific guides (33). However, these measurements were taken in dry cadaver bones, and there could be a discrepancy between these results and the realistic clinical accuracy as, in most cases, soft tissue envelope will block the vision when checking for the fit of patient-specific guides in the actual surgery setting (22). Promising results regarding the accuracy of three-dimensional corrective osteotomies of the forearm, especially for closed wedge osteotomies, have been reported by authors, such as less postoperative rotational deformity and less residual translation (33). Nevertheless, open wedge osteotomies (8.3 ± 5.35°) showed significant higher residual rotational deformity when compared to closing wedge (3.47 ± 1.09°) osteotomies. Campe et al. reported that restoration of the radius alignment to within 5° angular deformity and 2 mm ulnar variance in patients with malunion of the distal radius could be achieved in only 40% of the patients with the conventional technique (34). In contrast, 3-D virtual planning of corrective osteotomies of the distal radius has been reported to reach significant improvements in 96% of the cases with restorations to within 5° or 2 mm of their normal values in a systematic review and meta-analysis, including 15 studies with a total of 68 patients (11). Further, ulnar variances of 0 ± 1 mm and precise angular reconstruction in all included patients with distal radius malunions were reported by Stockmans et al. with the 3-D virtual planning (22). As stated by the same workgroup, volar tilt was more difficult to correct than the radial inclination and ulnar variance, as there was a tendency of under-correction of the fragment orientation, which hinged around the intraarticular osteotomy line (22). Despite these strengths of this innovative technique, there are some limitations that need to be addressed. The technique must be learned, and cooperation with clinical engineers to understand and apply the simulation procedure is required. In this case, the tendency to achieve higher accuracy is directly connected to experience on both, surgeons' and clinical design engineering side (22). Nevertheless, as a better visualization and guidance are associated with the 3-D preoperative planning technique, and the results in the literature suggest a high acceptance of the applicability by surgeons, the acquired experience could lead to an increased application of this technique, especially in highly complex cases in which the surgeon might not take the risk of the conventional surgery (35). Furthermore, the three-dimensional-planned technique is more expensive than the conventional method. Although the 3-D planning technique has been implemented in most radiology departments, the special software and the working hours of the clinical engineer for the planning process need to be taken into account (36). Additionally, cooperation with the specialized commercial companies is often required for the 3-D printing process, as the 3-D technology is time-consuming and yet not fully adapted in all hospital settings (11, 36). The costs for the three-dimensional corrective osteotomy technique for upper extremities have been noted to range from $2,000 to $4,000 depending on patients' preoperative situation (30). In contrast, the virtual surgery planning can be done multiple times with different virtual trials if once learned from the clinical engineer and thus is done at no additional cost, if no 3-D bone models are constructed. Moreover, the virtual project can be used with navigation systems already implemented at the respective surgery department. However, the use of patient-specific surgical guides is reported to be more time-saving than computer navigation techniques, and navigation systems for upper extremity corrective osteotomies are not easily applicable because of size-limiting factors of tracking markers and instruments (22, 37). One more point that has to be taken into account is the virtual planning and production time of the patient-specific guides, which takes between 6 and 8 weeks, depending on the complexity of the malunions (11, 22). The prolonged time between diagnosis of the malunion and surgery date could impair the functional disturbance and therefore be prognostically relevant for the outcome. Another disadvantage is that CT scans of both forearms are required for the three-dimensional planning technique, resulting in a higher radiation burden for the patients. However, the radiation exposure from CT scanning is considerably reduced below the normal dose used for diagnostic purposes because of the lower volume of the upper extremity compared to the thorax, abdomen, and pelvis (18). Computer simulation systems have been introduced in which 3-D bone models constructed from low dose radiation systems reached the same level of accuracy to those constructed from normal radiation doses CT data for three-dimensional corrective osteotomies techniques (18). In the future, the accuracy of such systems could be improved, resulting in a lower required radiation burden for the patients and a more precise bone surface model with lower errors. Finally, as described above, more randomized controlled trials evaluating the outcome in comparison with conventional techniques are required to account for this innovative but more expensive technique. To the author's best knowledge, there is only one randomized controlled trial comparing the computer-assisted vs. non-computer-assisted preoperative planning technique with regards to the corrective osteotomy of forearm malunions (30). Thus, the authors call for more well-designed randomized controlled trials comparing the two-dimensional and three-dimensional preoperative planning techniques to provide sufficient evidence regarding the correction of complex forearm malunions in modern surgical therapy approaches.

Three-dimensional corrective osteotomy based on preoperative computerized planning and patient-specific surgical guides resulted in excellent results in this case series. The promising evidence from the current literature and our case series suggest that preoperative computer-assisted three-dimensional planning techniques for corrective osteotomies of complex upper extremity malunion could be the new gold standard in the future.

## Data Availability Statement

The original contributions presented in the study are included in the article/supplementary material, further inquiries can be directed to the corresponding author/s.

## Ethics Statement

The studies involving human participants were reviewed and approved by Local institutional review board at the University Medical Center Freiburg, Freiburg, Germany. The patients/participants provided their written informed consent to participate in this study. Written informed consent was obtained from the individual(s) for the publication of any potentially identifiable images or data included in this article.

## Author Contributions

BS and GL did the literature search and wrote the first draft of the manuscript. JZ performed the surgery of the three cases, designed the study, proofread the manuscript, and made corrections. RS and AV did the literature search, proofread the manuscript, designed figures, and edited the manuscript. All authors contributed to the article and approved the submitted version.

## Conflict of Interest

The authors declare that the research was conducted in the absence of any commercial or financial relationships that could be construed as a potential conflict of interest.
